# Influence of Depression on Pain and Disability in Patients with Chronic Low Back Pain after Physical Therapy: A Secondary Analysis of a Randomized Controlled Trial

**DOI:** 10.1155/2024/9065325

**Published:** 2024-04-01

**Authors:** Rui Wang, Meng-Si Peng, Yi-Zu Wang, Pei-Jie Chen, Xue-Qiang Wang

**Affiliations:** ^1^Department of Rehabilitation Medicine, The Second Affiliated Hospital and Yuying Children's Hospital of Wenzhou Medical University, Wenzhou, Zhejiang, China; ^2^Department of Sport Rehabilitation, Shanghai University of Sport, Shanghai, China; ^3^Department of Rehabilitation Medicine, Shanghai University of Medicine and Health Sciences Affiliated Zhoupu Hospital, Shanghai, China; ^4^The Second Affiliated Hospital of Hainan Medical University, Haikou, Hainan, China; ^5^Department of Rehabilitation Medicine, Shanghai Jiao Tong University Medical School Affiliated Ruijin Hospital, Shanghai, China; ^6^School of Rehabilitation Medicine, Wenzhou Medical University, Wenzhou, Zhejiang, China

## Abstract

**Background:**

Depressive complications in chronic pain are detrimental to rehabilitation. This study was aimed at determining the influence of the presence of depressive symptoms on the efficacy of physical therapy among participants with chronic low back pain (CLBP).

**Methods:**

Data was collected from a randomized controlled trial on 113 participants with CLBP. Participants were reallocated into the depressed or nondepressed groups based on the 50-cutoff point of the self-rating depression scale. All patients received 60 min sessions of physical therapy twice a week for 12 weeks. The primary outcome was back-related disability. Secondary outcomes included pain ratings, sleep quality, life quality, other psychological outcomes, and minimal clinically important differences. These outcomes were collected at baseline, 12, 26, and 52 weeks.

**Results:**

31 (27.4%) were accompanied by depressive symptoms. At 12 weeks, the initial depression score was only associated with anxiety score (*β* = 1.196 [0.531 to 1.860], *P* = 0.001) and depression score (*β* = 0.742 [0.200 to 1.284], *P* = 0.009) in the depressed group, but the initial depression score was associated with anxiety score (*β* = 0.409 [0.138 to 0.681], *P* = 0.004), depression score (*β* = 0.920 [0.658 to 1.184], *P* < 0.001), sleep quality (*β* = 0.108 [0.018 to 0.199], *P* = 0.020), and pain anxiety (*β* = 0.465 [0.034 to 0.897], *P* = 0.035) and negatively associated with life quality (*β* = −0.815 [−1.267 to −0.363], *P* = 0.001) in the nondepressed group.

**Conclusions:**

Physical therapy is effective to CLBP with depressive symptoms. A higher initial depression score may weaken the efficacy of physical therapy in the nondepressed group. Depressive complications may adversely influence intervention efficacy for CLBP. This trial is registered with ChiCTR1800016396.

## 1. Introduction

Low back pain (LBP) is a pain syndrome with high prevalence and huge burden faced by people of all ages worldwide, and it is the primary cause of global years lived with disabilities [1, 2]. The prevalence of LBP increased by nearly 52.8% from 1990 to 2017, reaching 577 million people [3]. Women and people aged 40-80 have a higher prevalence [2]. Most people experience multiple recurrences of LBP after the first onset, resulting in chronic LBP (CLBP) and a lifetime prevalence of approximately 40% [4]. People with CLBP often suffer from functional limitations and comorbid conditions, such as insomnia, affective disorders, and depression [5]. Global surveys reported that individuals with CLBP have greater prevalence of psychiatric disorders than those without CLBP and have dysfunctional pain coping style [6, 7]. The prevalence of lifetime psychiatric disorders in CLBP can reach 74% [6]. Depression is the most common complication diagnosis of LBP, and the prevalence ranges from 4% to 18% [8, 9]. Women with musculoskeletal pain have a higher prevalence of severe depression [10]. A systematic review demonstrated that depressive symptoms at baseline are associated with worse outcomes for LBP at follow-up, and depression shows an effect in the direction of disadvantage [11]. Previous studies also reported that the association between chronic pain and depression is often a complicated vicious circle [12, 13]. Depressive complications in chronic pain tend to aggravate the condition and are detrimental to rehabilitation [12]. Therefore, the management of CLBP should consider the influence of biopsychosocial factors [14].

International guidelines and high-quality studies have indicated the benefits of different exercises for CLBP [15–17], and appropriate physical activities are also beneficial to mental health [18]. In our randomized controlled trial (RCT), a 12-week therapeutic aquatic exercise remarkably improved pain and disability for participants with CLBP compared with physical modality therapy [19]. However, some of the included participants reported depressive symptoms at baseline. Thus, the characteristics of changes in outcomes must be further analyzed to understand the influence of depression on the efficacy of interventions.

## 2. Methods

### 2.1. Study Design and Study Population

**Figure 1:**
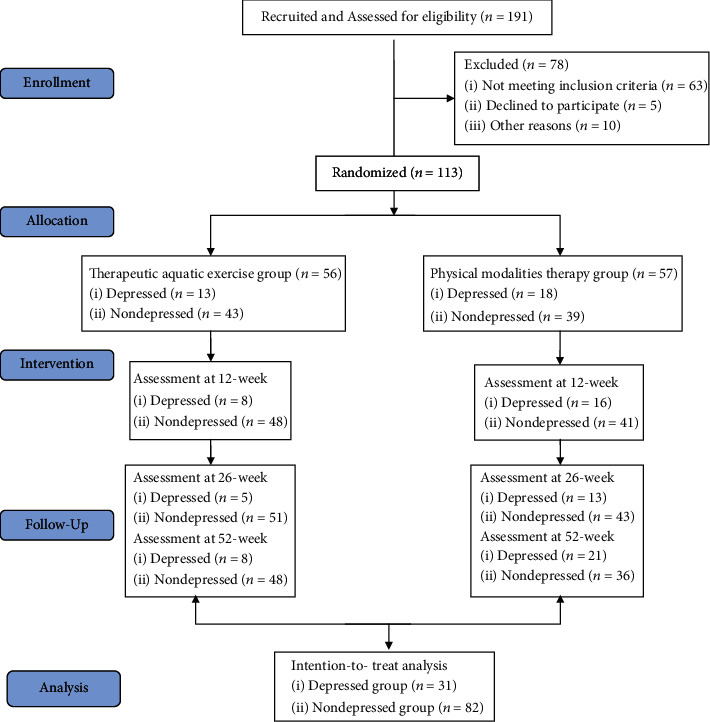
CONSORT (Consolidated Standards of Reporting Trials) flowchart.

The study conducted a secondary analysis of a 12-week two-arm RCT with a 52-week follow-up comparing the outcomes of physical therapy (i.e., therapeutic aquatic exercise and physical modality therapy) among participants with CLBP with or without depression, thereby exploring the characteristics of the influence of depression on the curative effect of interventions. The trial was completed on March 17, 2020, and the main results were published on January 7, 2022 [19]. Secondary data analysis was performed from July 4, 2022, to September 30, 2022. Detailed information of the trial registration and protocol is available elsewhere (Supplement 1) [19]. In this secondary analysis, we reclassified participants into the depression group with a score of 50 or higher and the CLBP without depression group with a score of less than 50 based on the score evaluated by the self-rating depression scale at baseline [20].  Figure 1 shows the Consolidated Standards of Reporting Trials (CONSORT) flowchart. The primary outcome for CLBP was back-related disability evaluated by the Roland-Morris disability questionnaire (RMDQ), in which the cutoff point of the minimal clinically important difference (MCID) is specified as a reduction of 3 points from baseline [21].

Briefly, participants were enrolled: aged 18 to 65 years, had LBP at least half the time in the last 6 months, a severe pain score of 3 or greater measured by the numerical rating scale (NRS) [22], and willingly participated in the trial with a written informed consent. The main exclusion criteria were as follows: signs of having specific causes of LBP (e.g., spondylolisthesis, spinal stenosis, and spinal tumor), having cognitive impairment, having serious mental illness, having red flags of serious or unstable chronic diseases, having aquatic exercise contraindications, receiving regular LBP exercise intervention or treatment in the past 6 months, addicted to alcohol or medicine, and pregnant or lactating. The participants were allowed to withdraw the trial because of unexpected serious conditions or side effect caused by physical therapy.

The Ethics Committee of the Shanghai University of Sport in China approved the trial (number: 2018042). The intervention of aquatic exercise was performed in the natatorium of the Shanghai University of Sport, and the physical modality therapy was performed in the Center of Sports Medicine and Rehabilitation, Shanghai Shangti Orthopaedic Hospital, Shanghai, China. The study complies with the CONSORT reporting guideline.

### 2.2. Randomization and Masking

After screening, 56 participants received therapeutic aquatic exercise and 57 received physical modality therapy by randomly drawing lots in numbered opaque envelopes. Each envelope contained a number drawn through a random number table. Researchers who participated in randomization and outcome collection were masked to the intervention allocation and hypotheses. Participants could not be masked because of their informed consent rights.

### 2.3. Interventions

In the second analysis, we reallocated the participants into the depressed group and nondepressed group. Therefore, both groups may have received therapeutic aquatic exercise or physical modality therapy. The aquatic exercise included 10 min of warm-up, 40 min of aquatic movements, and 10 min of relaxation. Aquatic movements included abdominal bracing, pressing water in different directions (vertical, lateral, and slant), straight leg raising, treading water, and running in deep water. The aquatic exercise was carried out in a group of 8–9 people. The physical modality therapy included infrared radiation therapy and transcutaneous electrical nerve stimulation. Both modalities were placed at points of LBP, and each lasted 30 min. The interventions were completed for 60 min each session, twice a week for 12 weeks. Details of the interventions have also been published [19].

### 2.4. Variables

The variables for the second analysis were collected at baseline, 12, 26, and 52 weeks. Demographic characteristics included age, height, weight, body mass index, history of LBP, levels of physical activities, sedentary time, smoking, medication use, and types of intervention. As the primary outcome, the RMDQ consists of 24 questions involving different daily activities. The participants answer “yes” (1 point) or “no” (0 point) to each question. A poorer back function is related to a higher score. The RMDQ is one of the most common scales to quickly assess lumbar function and has an excellent test-retest reliability [23]. The secondary outcomes included self-reported pain intensity (i.e., severe, average, and current NRS scores), quality of life measured by the 36-item short-form health survey [24], quality of sleep measured by the Pittsburgh sleep quality index [25], anxiety measured by the self-rating anxiety scale [26], depression measured by the self-rating depression scale [27], pain anxiety measured by the pain anxiety symptom scale [28], phobia of activity or reinjury measured by the Tampa scale for kinesiophobia [29], fear-avoidance belief measured by the fear-avoidance beliefs questionnaire [30], MCID in back-related disability [31], and MCID in pain intensity specified as a reduction of two points or more [31]. Detailed methods for the outcomes are available from elsewhere [19].

### 2.5. Statistical Analysis

Intention-to-treat analysis was performed in the secondary analysis. IBM SPSS Statistics 20.0 (SPSS Inc., Chicago, USA) and GraphPad Prism 9.0.0 (GraphPad Software, LLC) were used to analyze data and make diagrams. Categorical variables were represented by number (%), and continuous numerical variables were represented by mean (standard deviation). In all analyses, *P* value < 0.05 was considered statistically significant based on two-tailed tests.

At baseline, categorical variables were compared between the depressed group and the nondepressed group using *χ*^2^ tests. Normally distributed continuous numerical variables were analyzed using unpaired *t* tests, and nonnormally distributed variables were analyzed using the Mann–Whitney *U*-tests. *χ*^2^ tests were also used to compare proportion differences of the MCIDs in pain and disability between the depressed group and the nondepressed group. Repeated measure analysis of variance as supplementary analyses was performed to compare the outcomes between the two groups after interventions and at follow-up.

Adjusted multivariable linear regression analyses calculated coefficients (*β*) with 95% confidence intervals (CIs) to ascertain the influence of depression on continuous outcome variables after physical therapy and at 52-week follow-up. The covariates included gender, age, height, duration of LBP, levels of physical activity, smoking, sedentary time, and medication use. Moreover, adjusted binary logistic regression analyses calculated odds ratios (ORs) with 95% CIs to ascertain the association between depression score at baseline and MCIDs in pain and disability at 12 and 52 weeks. The covariates included gender, body mass index, and levels of physical activities. Success in achieving MCIDs was considered to be associated with an OR greater than 1.00.

## 3. Results

Among the 113 participants included in the trial, 31 (27.4%) had CLBP accompanied by depressive symptoms, and they were reallocated to the depressed group. The remaining 82 (72.6%) participants were included in the nondepressed group. The number of participants with depression decreased to 24 (21.2%) after the 12-week intervention and 18 (15.9%) at the 26-week follow-up, and the number rebounded to 29 (25.7%) at the 52-week follow-up (Figure 1). No significant differences in demographic characteristics were observed between the two groups (Table 1).

**Figure 2:**
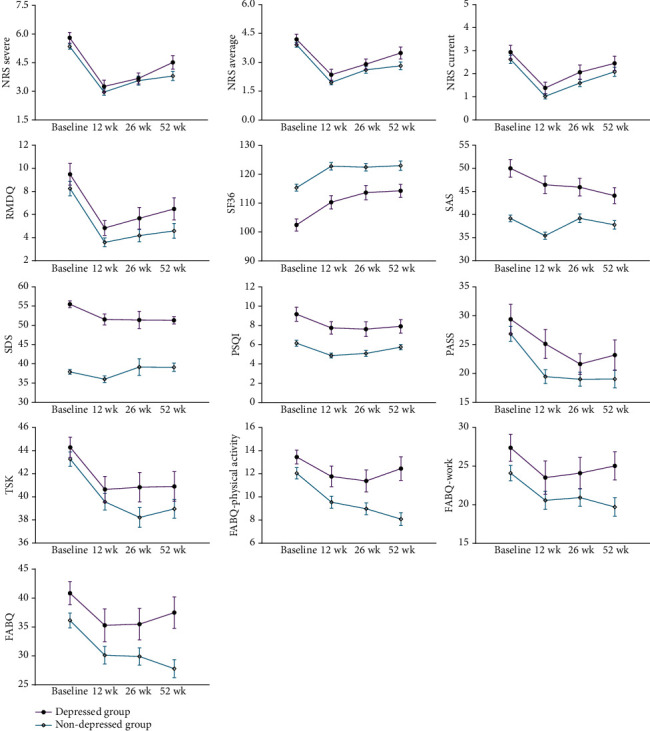
Improvement trend of outcomes stratified by depression at 12, 26, and 52 weeks.

The improvement trend of outcomes at 12, 26, and 52 weeks is shown in Figure 2. Both CLBP with or without depression groups showed varying degrees of improvement in pain intensity, back-related disability, anxiety, depression, sleep quality, life quality, and pain-related psychological outcomes after the 12-week physical therapy. Detailed comparisons of outcomes at 12, 26, and 52 weeks in the CLBP with or without depression groups are provided in Supplement 2. Briefly, both groups got improvements in pain and disability after the physical therapy, and the effect was maintained for at least 26 weeks. However, no differences were shown between groups. Thus, the physical therapy seemed to bring equivalent efficacy to both groups. The depressed group achieved better results in anxiety and depression than the nondepressed group, but the opposite was true for other pain-related psychological outcomes, such as pain anxiety, phobia of activity or reinjury, and fear-avoidance belief. Sleep quality and life quality were remarkably improved in both groups after physical therapy and at the follow-up phases. No difference was shown in the proportion of participants achieving MCIDs in pain and disability between the depressed group and the nondepressed group, and the proportion showed a decreasing trend during the follow-up phases (Supplement 3).

In the depressed group, the initial depression score was not linearly correlated with the changes in pain, disability, pain-related psychological outcomes, sleep quality, and life quality after the physical therapy, but a higher initial depression score was significantly correlated with a higher anxiety score (*β* = 1.196, 95%CI = 0.531 to 1.860, *P* = 0.001) and a higher depression score (*β* = 0.742, 95%CI = 0.200 to 1.284, *P* = 0.009) at 12 weeks. At the 52-week follow-up, only more severe NRS score was correlated with a higher initial depression score (*β* = 0.150, 95%CI = 0.012 to 0.287, *P* = 0.034) (Table 2). In the nondepressed group, the initial depression score was not linearly correlated with the changes in pain, disability, phobia of activity or reinjury, and fear-avoidance belief at 12 weeks. Moreover, a higher initial depression score was positively correlated with a higher anxiety score (*β* = 0.409, 95%CI = 0.138 to 0.681, *P* = 0.004), a higher depression score (*β* = 0.920, 95%CI = 0.658 to 1.184, *P* < 0.001), lower sleep quality (*β* = 0.108, 95%CI = 0.018 to 0.199, *P* = 0.020), and worse pain anxiety (*β* = 0.465, 95%CI = 0.034 to 0.897, *P* = 0.035), whereas it was negatively correlated with better life quality (*β* = −0.815, 95%CI = −1.267 to −0.363, *P* = 0.001) at 12 weeks. At the 52-week follow-up, the initial depression score was also significantly correlated with the changes in pain, anxiety, depression, sleep quality, life quality, and other psychological outcomes (Table 2).

In adjusted logistical regression models, the factors associated with the success in achieving MCIDs in pain and disability are presented in Table 3. The initial depression score was not correlated with MCID in disability at 12 and 52 weeks, either in the total sample or in the grouped sample. Further stratified analyses in the 12-week found that gender and age were significant moderators associated with MCID in disability. In the total sample and the depressed group, male (OR = 0.360, 95%CI = 0.155 to 0.836, *P* = 0.017; OR = 0.079, 95%CI = 0.007 to 0.851, *P* = 0.036, respectively) had less chance of achieving significant back function improvement. In the total sample and nondepressed group, the chance of achieving MCID in disability increased by 6.6% (OR = 1.066, 95%CI = 1.017 to 1.115, *P* = 0.007) and 7.4% (OR = 1.074, 95%CI = 1.013 to 1.140, *P* = 0.017) with each one-unit increase in age. Only the type of intervention was a significant moderator associated with MCID in disability in the 52-week regression models. Participants who accepted the aquatic exercise had more chance of achieving MCID in disability in the total sample (OR = 3.328, 95%CI = 1.525 to 7.261, *P* = 0.003) and the nondepressed group (OR = 3.709, 95%CI = 1.474 to 9.334, *P* = 0.005). With regard to pain, the chance of achieving MCID in the average pain increased by 11.5% (OR = 1.115, 95%CI = 1.018 to 1.221, *P* = 0.019) with each one-unit increase in initial depression score in the nondepressed group only at the 52-week follow-up.

## 4. Discussion

12-week physical therapy improved outcomes to varying degrees in the depressed group and nondepressed group, and more than half of the MCIDs in pain and disability were achieved in both groups without significant difference. A higher depression score at baseline likely attenuated the effect of physical therapy on outcomes in the nondepressed group, and physical therapy was likely more effective in achieving MCIDs in pain and disability in this group. The quantitative results of this secondary analysis indicated that depression at baseline may adversely influence the prognosis and outcomes of LBP. The results are consistent with the conclusions of a systematic review of 17 articles [11].

Depression may increase the likelihood of developing chronic pain and disability [32]. Compared with patients without depression, CLBP patients with depression have higher pain ratings and worse life quality, as well as lower work productivity [33]. Meanwhile, a growing number of studies have shown that depressive symptoms negatively influence the outcomes in the course of recovery [11]. Depression had a small-to-large impact on disability and a moderate-to-large impact on pain in patients who seek physical therapy for musculoskeletal pain [10]. Melloh et al. found that patients with acute LBP who initially had depressive symptoms had poor functional recovery after 3 and 6 months [34]. In addition, Wang et al. showed that depression seemed to influence the success of multidisciplinary pain therapy in patients with CLBP and depression achieving a comparable or even greater degree of improvement on clinical outcomes than patients with CLBP alone [35]. Our results showed comparable effects in the depressed and nondepressed groups after physical therapy, but the latter seemed to achieve more lasting success on pain relief and disability improvement at follow-up.

At present, the exact cause of the relationship between CLBP and depression remains to be elucidated. On the one hand, people with CLBP have hypersensitive pain perception and dysfunction in coping with pain, which more likely contribute to unpleasant emotional experiences [6, 36, 37]. On the other hand, depression is characterized by physical and emotional suffering; thus, the correlation between chronic pain and depression is bidirectional [38]. A cross-sectional study of 15,231 individuals found that the intensity of pain partially influenced the interaction between chronic disease and depression [39]. Poor psychological coping strategies may also play a mediating role [40]. Based on the cognitive-behavioral model, depression is a consequence of long-term pain, and multiple cognitive variables can modulate the association between pain and depression [41]. Coronado et al. demonstrated that fear of movement and pain self-efficacy mediated the effects of physical therapy on disability and physical health after spine surgery [42]. Joyce et al. found that improvements in perceived stress at 12 weeks positively mediated improvements in disability at 52 weeks after physical therapy in patients with CLBP [43]. From the neurobiological perspective, the considerable overlaps between chronic pain- and depression-induced neuroplasticity changes exist [12]. Studies demonstrated that CLBP led to brain structural and functional remodeling, including the anterior cingulate gyrus and prefrontal cortex, which are also the impaired regions in depression [44–46]. CLBP and depression have shared neurotransmitters such as serotonin involved in the descending pain inhibitory system and emotion regulation [47]. Increased proinflammatory cytokines also underlie the occurrence of both comorbidities [48, 49]. Moreover, CLBP and depression should have shared pharmacotherapy mechanisms, as evidenced by the recommendations on the pharmacological management of CLBP [50, 51].

Although depression at baseline tends to hinder the efficacy of interventions in the rehabilitation course of LBP, this study demonstrated that physical therapy can be conducive to CLBP patients with depression. In addition, the multimodal integration management of CLBP is worth considering [15]. Low-to-moderate evidence showed that exercise can improve pain and back-related disability compared with electrotherapy alone or other conservative interventions [16]. Furthermore, cognitive behavioral therapy, mindfulness, and self-management strategies are beneficial for CLBP patients with psychological problems such as low mood and negative pain beliefs [52]. Recently, Ashar et al. reported that pain reprocessing therapy aiming to change patients' beliefs about pain provided remarkable pain relief in patients with CLBP compared with placebo and usual care, and the effect was maintained at 1-year follow-up [53]. Longitudinal functional magnetic resonance imaging indicated that pain reprocessing therapy reduced responses to evoked back pain in the prefrontal and anterior midcingulate cortex, indicating a potential benefit for CLBP patients with depression [53]. Therefore, a combination of various treatments is more valuable for patients with CLBP accompanied by depression. Similarly, early screening for depression complications in patients with CLBP is important.

## 5. Limitations

The secondary analysis has several limitations. First, quite a few participants included in this trial were younger and had low self-reported pain intensity; thus, the generalizability of the results is limited. Second, the definition of MCIDs in pain and disability is heterogeneous in the previous studies, which could make differential results. Third, the sample size was smaller in the depressed group. The benefits of therapeutic aquatic exercise and physical modality therapy for a larger sample of participants with CLBP and depression are worth exploring in the future. Fourth, participants randomly accepted therapeutic aquatic exercise or physical modality therapy alone, but the combination of the two may be more beneficial for CLBP patients with depression.

## 6. Conclusions

In this study, 12-week physical therapy had a comparable effect on pain and disability in participants with CLBP with or without depressive symptoms. However, a higher initial depression score likely weakened the efficacy of physical therapy on outcomes in participants without depressive symptoms. This secondary analysis supported the efficacy of physical therapy in participants with CLBP and the characteristics of the influence of the presence of depressive symptoms on the efficacy of intervention.

## Figures and Tables

**Table 1:** Characteristics of study population at baseline stratified by depression.

Characteristic	CLBP with depression (*n* = 31)	CLBP without depression (*n* = 82)	*χ* ^2^/*z*/*t*	*P*
Gender, no. (%)				
Male	18 (58.1)	36 (43.9)	1.808	0.209
Female	13 (41.9)	46 (56.1)		
Age (year)	31.13 (11.13)	30.91 (11.65)	-0.097	0.923
Height (cm)	169.43 (7.64)	167.44 (8.52)	1.142	0.256
Weight (kg)	66.89 (11.83)	65.01 (13.71)	0.675	0.501
Body mass index (kg/m^2^)	23.26 (3.65)	22.99 (3.40)	-0.315	0.753
Years with CLBP (year)	6.67 (5.46)	6.78 (6.95)	-0.683	0.495
Siting time (hour)	6.39 (2.85)	6.29 (3.08)	-0.003	0.997
Moderate-intensity physical activities, no. (%)				
<150 min	10 (32.3)	27 (32.9)	0.056	0.972
150~300 min	9 (29.0)	22 (26.8)		
≥300 min	12 (38.7)	33 (40.2)		
High-intensity physical activities, no. (%)				
<75 min	12 (38.7)	40 (48.8)	1.461	0.482
75~150 min	10 (32.3)	18 (22.0)		
≥150 min	9 (29.0)	24 (29.3)		
Smoking, no. (%)				
Yes	7 (22.6)	13 (15.9)	0.699	0.417
No	24 (77.4)	69 (84.1)		
Medication, no. (%)				
Yes	20 (64.5)	62 (75.6)	1.391	0.247
No	11 (35.5)	20 (24.4)		
Interventions, no. (%)				
Aquatic exercise	13 (41.9)	43 (52.4)	0.993	0.400
Physical modalities	18 (58.1)	39 (47.6)		

Abbreviation: CLBP: chronic low back pain. Note: the presence of depressive symptoms is based on the cutoff of the self-rating depression scale. Patients with ≥50 points were divided into the depressed group; otherwise, they were nondepressed group.

**Table 2:** Influence of depression on the continuous outcomes at 12 and 52 weeks.

	CLBP with depression group			CLBP without depression group		
	Adjusted *β*	95% CI	*P* value	Adjusted *β*	95% CI	*P* value
Outcomes after the 12-week intervention						
RMDQ	-0.150	-0.418 to 0.118	0.262	0.140	0.000 to 0.281	0.051
NRS severe	-0.005	-0.147 to 0.137	0.941	0.035	-0.027 to 0.096	0.264
NRS average	0.020	-0.102 to 0.142	0.743	0.030	-0.017 to 0.077	0.208
NRS current	0.023	-0.084 to 0.130	0.662	-0.011	-0.055 to 0.034	0.637
SF36	-0.155	-1.135 to 0.825	0.749	-0.815	-1.267 to -0.363	0.001^a^
SAS	1.196	0.531 to 1.860	0.001^a^	0.409	0.138 to 0.681	0.004^a^
SDS	0.742	0.200 to 1.284	0.009^a^	0.921	0.658 to 1.184	<0.001^a^
PSQI	0.239	-0.023 to 0.502	0.073	0.108	0.018 to 0.199	0.020^a^
PASS	0.541	-0.502 to 1.583	0.297	0.465	0.034 to 0.897	0.035^a^
TSK	-0.215	-0.686 to 0.257	0.360	0.169	-0.097 to 0.436	0.210
FABQ-physical activity	-0.143	-0.521 to 0.234	0.444	0.126	-0.068 to 0.320	0.201
FABQ-work	-0.246	-1.162 to 0.671	0.588	0.115	-0.322 to 0.552	0.602
FABQ	-0.389	-1.581 to 0.803	0.510	0.241	-0.326 to 0.807	0.401
Outcomes after the 52-week follow-up						
RMDQ	-0.203	-0.534 to 0.129	0.220	0.167	-0.069 to 0.402	0.162
NRS severe	0.150	0.012 to 0.287	0.034^a^	0.069	-0.019 to 0.157	0.124
NRS average	0.064	-0.064 to 0.192	0.315	0.072	0.000 to 0.144	0.049^a^
NRS current	0.082	-0.047 to 0.212	0.204	0.092	0.022 to 0.163	0.011^a^
SF36	0.244	-0.707 to 1.195	0.604	-0.996	-1.553 to -0.439	0.001^a^
SAS	0.272	-0.460 to 1.005	0.453	0.753	0.455 to 1.051	<0.001^a^
SDS	0.186	-0.729 to 1.100	0.681	1.008	0.669 to 1.347	<0.001^a^
PSQI	0.166	-0.126 to 0.458	0.254	0.180	0.085 to 0.274	<0.001^a^
PASS	0.417	-0.624 to 1.458	0.419	0.640	0.083 to 1.197	0.025^a^
TSK	0.288	-0.815 to 1.391	0.597	0.589	0.034 to 1.144	0.038^a^
FABQ-physical activity	-0.058	-0.506 to 0.389	0.792	0.281	0.080 to 0.481	0.007^a^
FABQ-work	-0.095	-0.847 to 0.657	0.797	0.026	-0.410 to 0.462	0.906
FABQ	-0.153	-1.309 to 1.003	0.788	0.296	-0.269 to 0.861	0.300

Abbreviations: CLBP: chronic low back pain; CI: confidence interval; NRS: numeric rating scale; RMDQ: Roland-Morris disability questionnaire; SF36: 36-item short-form health survey; SAS: self-rating anxiety scale; SDS: self-rating depression scale; PSQI: Pittsburgh sleep quality index; PASS: pain anxiety symptom scale; TSK: Tampa scale for kinesiophobia; FABQ: fear-avoidance beliefs questionnaire. ^a^Data statistically significant.

**Table 3:** Influence of depression on the minimal clinically important difference of pain and disability.

Variables	Adjusted logistic regression model after the 12-week intervention			Adjusted logistic regression model after the 52-week follow-up		
	Adjusted OR	95% CI	*P* value	Adjusted OR	95% CI	*P* value
RMDQ MCID						
Total	1.041	0.994 to 1.090	0.092	0.987	0.947 to 1.029	0.549
(i) Aquatic exercise	NA	NA	NA	3.328	1.525 to 7.261	0.003^a^
(ii) Male	0.360	0.155 to 0.836	0.017^a^	NA	NA	NA
(ii) Age	1.066	1.017 to 1.116	0.007^a^	NA	NA	NA
CLBP with depression group	1.196	0.859 to 1.664	0.289	0.994	0.857 to 1.153	0.936
(i) Aquatic exercise	NA	NA	NA	2.504	0.577 to 10.872	0.220
(ii) Male	0.079	0.007 to 0.851	0.036^a^	NA	NA	NA
(iii) Age	1.044	0.963 to 1.132	0.299	NA	NA	NA
CLBP without depression group	1.062	0.971 to 1.161	0.186	0.991	0.909 to 1.081	0.838
(i) Aquatic exercise	NA	NA	NA	3.709	1.474 to 9.334	0.005^a^
(ii) Male	0.564	0.215 to 1.480	0.244	NA	NA	NA
(iii) Age	1.074	1.013 to 1.140	0.017^a^	NA	NA	NA
NRS severe MCID						
Total	1.021	0.974 to 1.070	0.387	1.019	0.977 to 1.062	0.389
CLBP with depression group	0.905	0.765 to 1.070	0.242	1.161	0.936 to 1.441	0.174
CLBP without depression group	1.048	0.954 to 1.151	0.328	1.057	0.971 to 1.151	0.201
NRS average MCID						
Total	1.014	0.972 to 1.057	0.514	0.989	0.948 to 1.032	0.614
CLBP with depression group	0.922	0.757 to 1.122	0.417	0.870	0.702 to 1.078	0.202
CLBP without depression group	1.042	0.956 to 1.134	0.350	1.115	1.018 to 1.221	0.019^a^
NRS current MCID						
Total	1.016	0.977 to 1.057	0.432	1.020	0.977 to 1.064	0.371
CLBP with depression group	1.021	0.882 to 1.181	0.781	1.095	0.938 to 1.278	0.251
CLBP without depression group	1.029	0.948 to 1.116	0.497	1.047	0.957 to 1.146	0.316

Abbreviations: RMDQ: Roland-Morris disability questionnaire; MCID: minimal clinically important difference; CLBP: chronic low back pain; NRS: numeric rating scale; CI: confidence interval; NA: not applicable, and insufficient data to run the analysis. ^a^Data statistically significant.

## Data Availability

The data that support the findings of this study are available from the corresponding authors upon reasonable request.

## Supplementary Materials

Supplementary 1 Trial protocol.

Supplementary 2 Outcomes at 12, 26, and 52 weeks in the chronic low back pain with or without depression groups.

Supplementary 3 Differences of achieving clinical improvement in pain and disability stratified by depression.
