# Supplementary material for: Influence of Depression on Pain and Disability in Patients with Chronic Low Back Pain after Physical Therapy: A Secondary Analysis of a Randomized Controlled Trial
Source: Depress Anxiety. 2024 Apr 1;2024:9065325. doi: 10.1155/2024/9065325 (PMC11919046; doi:10.1155/2024/9065325)
Supplement: Supplementary 2 — Outcomes at 12, 26, and 52 weeks in the chronic low back pain with or without depression groups. [file 9065325.f2.docx]

Supplement 2 Outcomes at 12, 26, and 52 weeks in the depressed and non-depressed groups

| Outcomes | CLBP with depression  (n=31) | CLBP without depression  (n=82) | Between-group difference (95% CI) | *P* Value | F Value | *P* Value for Overall Group × Time Interaction |
| --- | --- | --- | --- | --- | --- | --- |
| RMDQ |  |  |  |  |  |  |
| Baseline | 9.48(5.27) | 8.26(5.71) | 1.23[-1.11,3.57] | 0.300 | 0.244 | 0.865 |
| 12 wk | 4.84(3.60) ^b^ | 3.60(3.48) ^b^ | 1.24[-0.23,2.71] | 0.097 |  |  |
| 26 wk | 5.68(5.22) ^b^ | 4.18(4.85) ^b^ | 1.49[-0.58,3.56] | 0.155 |  |  |
| 52 wk | 6.48(5.35) ^b, c^ | 4.59(5.84) ^b, c^ | 1.90[-0.49,4.28] | 0.118 |  |  |
| NRS severe |  |  |  |  |  |  |
| Baseline | 5.81(1.58) | 5.35(1.29) | 0.45 [-0.12, 1.03] | 0.121 | 1.054 | 0.372 |
| 12 wk | 3.26(1.86) ^b^ | 2.96(1.50) ^b^ | 0.30[-0.38, 0.97] | 0.387 |  |  |
| 26 wk | 3.68(1.62) ^b^ | 3.56(2.13) ^b, c^ | 0.12[-0.72, 0.95] | 0.783 |  |  |
| 52 wk | 4.52 (1.95) ^b, c, d^ | 3.81(2.16) ^b, c^ | 0.71[-0.17, 1.59] | 0.112 |  |  |
| NRS Average |  |  |  |  |  |  |
| Baseline | 4.19(1.47) | 3.91(1.17) | 0.28[-0.25,0.80] | 0.295 | 0.619 | 0.604 |
| 12 wk | 2.35(1.60) ^b^ | 1.95(1.15) ^b^ | 0.40[-0.14,0.94] | 0.141 |  |  |
| 26 wk | 2.06(1.73) ^b^ | 1.60(1.43) ^b, c^ | 0.29[-0.38,0.97] | 0.390 |  |  |
| 52 wk | 3.48(1.73) ^b, c, d^ | 2.82(1.80) ^b, c^ | 0.67[-0.08,1.41] | 0.078 |  |  |
| NRS Current |  |  |  |  |  |  |
| Baseline | 2.94(1.65) | 2.62(1.60) | 0.31[-0.36,0.99 | 0.359 | 0.054 | 0.984 |
| 12 wk | 1.39(1.41) ^b^ | 1.02(1.08) ^b^ | 0.36[-0.13,0.85] | 0.146 |  |  |
| 26 wk | 2.06(1.73) ^b, c^ | 1.60(1.43) ^b, c^ | 0.47[-0.17,1.10] | 0.147 |  |  |
| 52 wk | 2.45(1.75) ^b, c^ | 2.09(1.77) ^b, c, d^ | 0.37[-0.37,1.10] | 0.327 |  |  |
| SF36 |  |  |  |  |  |  |
| Baseline | 102.45(11.72) | 115.35(10.82) | -12.89[-17.52, -9.27] | <0.001 | 1.143 | 0.335 |
| 12 wk | 110.31(12.87) ^b^ | 122.78(11.79) ^b^ | -12.48[-17.53, -7.43] | <0.001 |  |  |
| 26 wk | 113.64(13.73) ^b^ | 122.44(11.94) ^b^ | -8.80[-14.00, -3.60] | 0.001 |  |  |
| 52 wk | 114.27(12.52) ^b^ | 122.99(14.51) ^b^ | -8.72[-14.57, -2.87] | 0.004 |  |  |
| SAS |  |  |  |  |  |  |
| Baseline | 50.00(10.62) | 39.17(6.53) | 10.83[7.55,14.11] | <0.001 | 3.024 | 0.033 ^a^ |
| 12 wk | 46.45(10.55) ^b^ | 35.38(6.88) ^b^ | 11.07[7.72,14.43] | <0.001 |  |  |
| 26 wk | 45.94 (10.76) ^b^ | 39.09(8.53) ^c^ | 6.85[3.01,10.69] | 0.001 |  |  |
| 52 wk | 44.10(9.70) ^b^ | 37.69(8.24) ^c^ | 6.41[2.80,10.03] | 0.001 |  |  |
| SDS |  |  |  |  |  |  |
| Baseline | 55.48(4.98) | 37.91(5.38) | 17.57[15.36,19.77] | <0.001 | 2.994 | 0.034 ^a^ |
| 12 wk | 51.55(8.01) ^b^ | 35.88(8.07) ^b^ | 15.67[12.31,19.04] | <0.001 |  |  |
| 26 wk | 51.39(12.56) ^b^ | 39.02(9.71) ^c^ | 12.38[7.96,16.78] | <0.001 |  |  |
| 52 wk | 51.32(12.03) ^b^ | 38.99(9.83) ^c^ | 12.33[7.96,16,71] | <0.001 |  |  |
| PSQI |  |  |  |  |  |  |
| Baseline | 9.16(4.08) | 6.15(2.81) | 3.01[1.67,4.34] | <0.001 | 0.627 | 0.599 |
| 12 wk | 7.74(3.64) ^b^ | 4.88(2.26) ^b^ | 2.86[1.73, 3.99] | <0.001 |  |  |
| 26 wk | 7.61(4.23) ^b^ | 5.10(2.84) ^b^ | 2.52[1.15,3,89] | <0.001 |  |  |
| 52 wk | 7.90(3.91) ^b^ | 5.74(2.48) ^c, d^ | 2.16[0.93,3.39] | 0.001 |  |  |
| PASS |  |  |  |  |  |  |
| Baseline | 29.39(14.41) | 26.85(16.66) | 2.53[-2.67,7.74] | 0.337 | 0.740 | 0.530 |
| 12 wk | 25.13(13.93) | 19.45(10.75) ^b^ | 5.68[0.79,10.56] | 0.023 |  |  |
| 26 wk | 21.65(9.95) ^b^ | 19.01(10.85) ^b^ | 2.63[-1.80,7.07] | 0.242 |  |  |
| 52 wk | 23.19(14.72) ^b^ | 19.05(13.89) ^b^ | 4.15[-1.76,10.04] | 0.167 |  |  |
| MTSK |  |  |  |  |  |  |
| Baseline | 44.29(4.95) | 44.27(5.67) | 1.02[-1.27,3.13] | 0.379 | 0.613 | 0.608 |
| 12 wk | 40.65(6.27) ^b^ | 39.57(6.52) ^b^ | 1.07[-1.62,3.77] | 0.432 |  |  |
| 26 wk | 40.84(7.13) ^b^ | 38.22(7.77) ^b, c^ | 2.62[-0.56,5.80] | 0.105 |  |  |
| 52 wk | 40.90(7.14) ^b^ | 38.97(7.36) ^b^ | 1.94 [-1.11,4.99] | 0.210 |  |  |
| FABQ-physical activity |  |  |  |  |  |  |
| Baseline | 13.45(3.29) | 12.05(4.49) | 1.40[-0.35,3.16] | 0.116 | 2.836 | 0.041 ^a^ |
| 12 wk | 11.77(5.00) | 9.55(4.40) ^b^ | 2.23[0.21,4.24] | 0.030 |  |  |
| 26 wk | 11.39(5.29) ^b^ | 8.98(4.68) ^b^ | 2.41[0.38,4.44] | 0.020 |  |  |
| 52 wk | 12.45(5.66) | 8.09(5.05) ^b, c^ | 4.37[2.18,6.55] | <0.001 |  |  |
| FABQ-work |  |  |  |  |  |  |
| Baseline | 27.39(9.81) | 24.10(8.99) | 3.29[-0.56,7.14] | 0.093 | 0.561 | 0.642 |
| 12 wk | 23.52(12.08) ^b^ | 20.57(10.61) ^b^ | 2.94[-1.66,7.55] | 0.208 |  |  |
| 26 wk | 24.10(11.32) | 20.93(10.24) ^b^ | 3.17[-1.24,7.58] | 0.157 |  |  |
| 52 wk | 25.03(10.14) | 19.71(10.83) ^b^ | 5.33[0.88,9.77] | 0.019 |  |  |
| FABQ |  |  |  |  |  |  |
| Baseline | 40.84(11.14) | 36.15(11.60) | 4.69[-0.10,9.49] | 0.055 | 1.272 | 0.284 |
| 12 wk | 35.29(15.75) ^b^ | 30.12(13.77) ^b^ | 5.17[-0.82,11.16] | 0.090 |  |  |
| 26 wk | 35.48(15.21) ^b^ | 29.90(13.58) ^b^ | 5.58[-0.28,11.45] | 0.062 |  |  |
| 52 wk | 37.48 (15.15) | 27.79(14.05) ^b^ | 9.69[3.69,15.69] | 0.002 |  |  |

Abbreviations: CLBP, Chronic Low Back Pain; CI, Confidence Interval; NRS, Numeric Rating Scale; RMDQ, Roland-Morris Disability Questionnaire; SF36, 36-item Short-Form Health Survey; SAS, Self-Rating Anxiety Scale; SDS, Self-rating Depression Scale; PSQI, Pittsburgh Sleep Quality Index; PASS, Pain Anxiety Symptoms Scale; TSK, Tampa Scale for Kinesiophobia; FABQ, Fear-Avoidance Beliefs Questionnaire.

^a^ Statistically significant interaction of time and group

^b^ Statistically significant difference compared to the data at baseline

^c^ Statistically significant difference compared to the data at 12-week

^d^ Statistically significant difference compared to the data at 26-week
