# Supplementary material for: Influence of Depression on Pain and Disability in Patients with Chronic Low Back Pain after Physical Therapy: A Secondary Analysis of a Randomized Controlled Trial
Source: Depress Anxiety. 2024 Apr 1;2024:9065325. doi: 10.1155/2024/9065325 (PMC11919046; doi:10.1155/2024/9065325)
Supplement: Supplementary 3 — Differences of achieving clinical improvement in pain and disability stratified by depression. [file 9065325.f3.docx]

Supplement 3 Differences in achieving clinical improvement in pain and disability

| MCIDs | CLBP with depression(n=31) | CLBP without depression(n=82) | χ^2^ | *P* value |
| --- | --- | --- | --- | --- |
| NRS severe MCID, No. (%) |  |  |  |  |
| 12 wk | 25 (80.65%) | 60 (73.17%) | 0.674 | 0.473 |
| 26 wk | 20 (64.52%) | 50 (60.98%) | 0.120 | 0.829 |
| 52 wk | 15 (48.39%) | 44 (53.66%) | 0.251 | 0.676 |
| NRS average MCID, No. (%) |  |  |  |  |
| 12 wk | 20 (64.52%) | 50 (60.98%) | 0.120 | 0.829 |
| 26 wk | 12 (38.71%) | 39 (47.56%) | 0.712 | 0.526 |
| 52 wk | 7 (22.58%) | 35 (42.68%) | 3.893 | 0.053 |
| NRS current MCID, No. (%) |  |  |  |  |
| 12 wk | 17 (54.84%) | 41 (50.00%) | 0.211 | 0.678 |
| 26 wk | 11 (25.48%) | 35 (42.68%) | 0.483 | 0.527 |
| 52 wk | 9 (29.03%) | 23 (28.05%) | 0.011 | 1.000 |
| RMDQ MCID, No. (%) |  |  |  |  |
| 12 wk | 21 (67.74%) | 51 (62.20%) | 0.299 | 0.584 |
| 26 wk | 19 (61.30%) | 48 (58.50%) | 0.071 | 0.790 |
| 52 wk | 15 (48.39%) | 47 (57.31%) | 0.724 | 0.395 |

Abbreviations: CLBP, Chronic Low Back Pain; NRS, Numeric Rating Scale; RMDQ, Roland-Morris Disability Questionnaire; MCID, Minimal Clinically Important Difference.
